# A longitudinal study on multiple frailty and its associations with depression and social participation in older adults

**DOI:** 10.1038/s41598-026-45343-1

**Published:** 2026-04-05

**Authors:** JuHee Lee, Jungah Park, Miji Kim, Sumi Lee, Chang Won Won

**Affiliations:** 1 College of Nursing, Mo-Im Kim Nursing Research Institute, Yonsei University, Seoul, Republic of Korea; 2 College of Nursing and Brain Korea 21 FOUR Project, Yonsei University, Seoul, Republic of Korea; 3 College of Nursing, CHA University, Pocheon, 11160 Republic of Korea; 4 Department of Health Sciences and Technology, College of Medicine, Kyung Hee University, Seoul, Republic of Korea; 5 Department of Nursing Science, Howon University, Gunsan, Republic of Korea; 6 Elderly Frailty Research Center, Department of Family Medicine, College of Medicine, Kyung Hee University, Seoul, Republic of Korea; 7 Department of Family Medicine, Kyung Hee University Medical Center, Seoul, Republic of Korea

**Keywords:** frailty, depression, social participation, health check-ups, balance confidence, fear or falling, Diseases, Health care, Medical research, Risk factors

## Abstract

To explore the changes in the physical, cognitive, and social frailty over a two-year follow-up and to identify the predictors associated with multiple frailty. This longitudinal cohort study utilized data from 2,534 recruited participants aged ≥ 70 years from the Korean Frailty and Aging Cohort Study (2016–2019). From baseline to follow-up, the percentages of physical, cognitive, and social frailty decreased from 3.6% to 3.0%, 2.9% to 2.1%, and 18.3% to 10.5%, respectively. Factors that influenced multiple frailty were taking more than five medications increased the risk of physical and cognitive frailty (odds ratio [OR] = 1.34, 95% confidence interval [CI]: 1.18–1.53, OR = 2.27, 95% CI: 1.53–3.38, respectively). The absence of national health check-ups increased the risk of physical and social frailty by 1.31 times and 1.30 times (95% CI: 1.11–1.54, 95% CI: 1.04–1.63, respectively). Depression and lower social participation emerged as key risk factors across multiple frailty. This research comprehensively understands modifiable and non-modifiable factors contributing to multiple frailty. By identifying depression and social participation as the most significant predictors across multiple frailty, this study highlights the urgent need for integrated interventions to prevent and manage multiple frailty.

**Supplementary Information:**

The online version contains supplementary material available at 10.1038/s41598-026-45343-1.

## Introduction

Frailty in older adults is commonly characterized by physical deterioration^1^. Physical frailty contributes significantly to health-related outcomes, including falls, bone fractures, hospitalization, and mortality^2^. Recent research highlights the need for a multidimensional framework that includes cognitive and social vulnerabilities alongside physical decline^3–7^. While multiple frailty has gained attention as a key concept, consensus on its definition remains limited^5,6^. The concept is still taking shape in academic discourse. It represents a shift toward a more person-centered approach that prioritizes an older adult’s functional maintenance for independent living^4–7^. Rather than viewing frailty only as biological aging or a set of physical measures, we conceptualize it as a multidimensional state essential for autonomy within the community^4^. We therefore operationalize multiple frailty as the co-occurrence of physical, cognitive, and social deficits. This integrated approach offers a more complete assessment of vulnerability and supports the development of targeted interventions in aging populations.

Focusing solely on physical aspects overlooks other significant vulnerabilities that affect aging populations. Multiple frailty is associated with negative health outcomes, such as dementia, admission to long-term care, or higher mortality^3,4^. Recent studies emphasize the multiple frailty for a more integrated approach that considers not only physical frailty but also cognitive and social frailty^4–7^. Frailty is also accompanied simultaneously by physical, cognitive, and social aspects.

Previous research emphasizes the dynamic nature of frailty, highlighting it as a non-static condition that can deteriorate, stabilize, or recover^8,9^. Physical frailty is recognized as a dynamic health state associated with advanced age, polypharmacy, poor nutrition, lack of physical activity, and depression^8,10^. Cognitive frailty is linked to low educational attainment, high levels of depressive symptoms, poor nutritional status, and activity balance confidence^9,11,12^. Social frailty is predicted by hearing loss, insufficient social support, and low engagement in social activities^13,14^.

The information available about changes in multiple frailty is still limited, so it is necessary to understand the timing of interventions to prevent or delay multiple frailty. Geriatric experts should focus on regular assessment and timely intervention to design health care plans that can delay or improve frailty before it becomes an irreversible. A substantial body of research has been identified regarding factors associated with a particular frailty. Physical, cognitive, and social frailty are influenced by both distinct and overlapping factors. These factors need to be categorized into those affecting specific frailty or those affecting multiple frailty. As evidence has shown that multiple frailty are linked to negative health outcomes in later life^3,4^, incorporating related factors into both research and clinical settings has become increasingly important in the super-aged society. Understanding these predictors is essential for the developing multiple frailty interventions and informing integrated multiple frailty care. Ultimately, this multidimensional framework aims to enhance functional resilience in older adults by identifying modifiable predictors.

The objectives were to investigate the changes over time in multiple frailty: physical, cognitive, and social frailty, and to identify factors that predict multiple frailty.

## Methods

### Design

This research conducted a longitudinal survey design in a secondary analysis of datasets from the Korean Frailty and Aging Cohort Study (KFACS). The KFACS datasets includes collected information aimed to examine various aspects such as demographic characteristics, physical health, psychological status, and frailty among older adults in South Korea^15^. The KFACS consisted of a start phase from 2016 to 2017, and a follow-up phase from 2018 to 2019. The KFACS is a reliable frailty of older adults aged 70 years or older, using large-scale, stratified sampling^15^. Further details about the KFACS are available in a previously published report^15^.

### Setting and Participants

The inclusion criteria were as follows: (1) completion of all three frailty assessments—physical, cognitive, and social—at both baseline and the two-year follow-up; and (2) availability of baseline demographic and health-related variables. All participants were included regardless of their initial frailty status (non-frail, pre-frail, or frail) at baseline, provided they met these follow-up requirements.

**Fig. 1:**
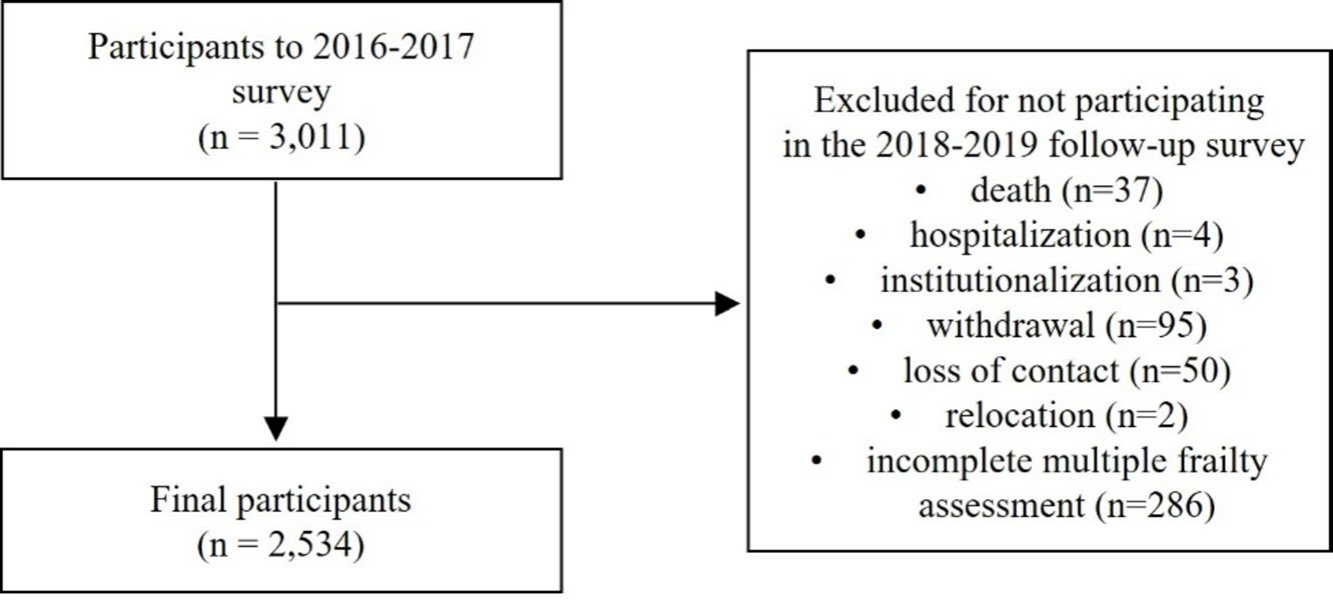
Flow of the selection process for this study sample.

A total of 3011 individuals participated at baseline. Among the 477 participants lost to follow-up, reasons included death (*n* = 37), hospitalization (*n* = 4), institutionalization (*n* = 3), withdrawal of participants (*n* = 95), relocation (*n* = 2), loss of contact (*n* = 50), and incomplete multiple frailty assessment (*n* = 286). Therefore, a total of 2,534 participants were finally included (Fig. 1).

### Instruments

In this study, the instruments used to measure multiple frailty, demographic and health-related factors are presented.

### Physical frailty

Physical frailty was assessed using the five components of the Fried frailty phenotype: weakness, exhaustion, weight loss, slowness, and low physical activity^8^. Weakness was evaluated with a handgrip dynamometer, measuring in kilograms^15^. Weakness was defined as handgrip strength below 18 kg in women and below 28 kg in men, based on the AWGS 2019 criteria^16^. Exhaustion was identified through positive responses to two questions: “Did you feel that everything was difficult?” and “Did you struggle to motivate yourself to do anything?” Unintentional weight loss was defined as a reduction in body weight of more than 4.5 kg over the past year. Slowness was manually measured by the time taken to walk more than 4 m and defined as being in the lowest 20% based on sex and height. Low physical activity was assessed using the International Physical Activity Questionnaire and defined as less than 494.65 kcal for males and less than 283.50 kcal for females^17^. The classification criteria identified frailty with three or more components, pre-frailty with one or two components, and non-frailty with none.

### Cognitive frailty

Cognitive frailty is defined as the presence of both cognitive impairment and physical frailty, excluding individuals diagnosed with dementia^18^. Physical frailty was characterized according to the Fried frailty phenotype. To ensure a comprehensive assessment of cognitive impairment, we utilized a diverse battery of tests, including the Mini-Mental State Examination in the Korean version of the CERAD assessment packet (MMSE-KC), the Frontal Assessment Battery (FAB), the Digit Span (DS), and the Trail Making Tests (TMT)^19^. We adopted a conservative definition of cognitive impairment based on age-, sex-, and education-adjusted normative values^20^. In this study, cognitive frailty was identified by the presence of at least three out of five physical frailty phenotypes, the absence of dementia diagnosis, and scores below a cutoff on one or more of four cognitive tests. This approach, utilizing various assessment tools, likely accounts for the relatively lower prevalence than previous studies, thereby minimizing false positives.

### Social frailty

Social frailty was assessed using five components: living alone, frequency of meeting friend(s), daily family contact, participation, and the presence of a person who shows love and affection^14^. Living alone was self-reported. The frequency of meeting or talking to friends was categorized as low if it occurred less than once a month^21,22^. Daily family contact was evaluated by asking respondents if they met or talked with family members (including relatives and neighbours) less frequently than daily^21^. Participation in community and social gatherings (such as senior centers, clubs, classes, and group meetings) was assessed by responses indicating participation once a month, occasionally, or never^21^. The presence of a person who shows love and affection was identified through the question “Is there someone available for you and who shows you love and affection?” and answered as “a little of the time” or “none.”^20^. The classification defined social frailty as having three or more components, social pre-frailty as having one or two, and social non-frailty as the absence.

### Demographic factors

We assessed the following demographic factors: age, sex, education (primary school or less, or middle school or higher), marital status (married including widow/widower and separated/divorced, or unmarried), and health insurance (workplace and local health insurance, medical aid).

### Health-related factors

Health literacy was assessed using the Brief Health Literacy Screen with three items. Responses were rated on a 5-point scale (1 = always to 5 = never)^23^. A total score ranging from 3 to 15 was calculated by summing all item responses, with lower scores indicating higher health literacy. A total score of 3 was classified as adequate health literacy^24^, while scores ranging from 4 to 15 were classified as inadequate health literacy. Comorbidities included ten physician-diagnosed diseases, including respiratory diseases, circulatory diseases, musculoskeletal diseases, cancer, infections, and mental disorders, and were evaluated on a scale of 0 to 10. The number of medications was dichotomized into two categories: four or fewer and five or more, based on medications taken continuously for more than three months. Hearing loss was measured by assessing hearing thresholds at specific frequencies: 500, 1000, 2000, and 4000 Hz using pure-tone audiometry and classified by the results for pure-tone average^14^. A higher average indicates greater hearing loss, meaning louder sounds are needed to hear. According to the criteria of the World Health Organization (2024)^25^, participants with the average of 35 dB or more were classified as hearing loss. Health check-ups were categorized based on whether participants had received a national health check-up in the past two years. Activity-specific balance confidence was defined as confidence in performing general daily activities without losing balance. It was measured using the Korean version of the Activity-specific Balance Confidence scale, with higher scores indicating greater balance confidence^26^. We assessed fear of falling (FOF) using responses to the question “Are you concerned about falling?” Participants’ answers were categorized into four levels: (1) “Not at all worried,” (2) “Slightly worried,” (3) “Quite worried,” and (4) “Very worried.” We then classified FOF based on these responses. Participants who responded with 1 were classified as no FOF, 2 as low FOF, and 3 or 4 as high FOF^27^. The Korean version of the Short Form Geriatric Depression Scale was used to assess depression, with higher scores indicating greater levels of depression^28^. Social participation was assessed by asking about involvement in various group activities, including social, religious, cultural, sports, civic, political, service, and learning groups. The scale ranged from 0 to 8, with higher scores indicating more active social participation. The activity environment was assessed using the International Physical Activity Questionnaire Environment Module (IPAQ-E), a self-report instrument measuring environmental factors associated with physical activity^29^. Higher scores indicated better physical activity environment.

### Data analysis

To explore the demographic and health-related factors based on multiple frailty at baseline, we calculated percentages, means, and standard deviations (SD) and performed t-tests and chi-square tests. To examine the associations between demographic and health-related factors and longitudinal multiple frailty assessments, binary and ordinal logistic regression models were employed within the framework of generalized estimating equations. Binary and ordinal logistic regressions produced odds ratios (ORs) and 95% confidence intervals (CIs). The proportion of missing data for key variables was low, ranging from 0.08% (education, marital status, and hearing loss) to 1.85% (health insurance). Given that missingness for all covariates was less than 2%, statistical analyses were performed using complete case analysis within the GEE framework^30^. This minimal amount of missing data is unlikely to have affected the overall results.

### Ethical consideration

This research utilized the KFACS data and was approved by the Institutional Review Boards of Kyung Hee University (No. 2015-12-103) and Howon University (No. 1041585-202412-HR-002-01). The data for our study used the KFACS, which targets community-dwelling adults aged 70 and over.

### Reporting Guidelines

This study was reported using the STROBE checklist, and the completed checklist is provided in Supplementary Table S1.

Large Language Model (LLM) technology was utilized for the English translation of the Korean manuscript. The authors reviewed and edited the entirety of the translated content for accuracy and appropriate scientific terminology.

## Results

### Participants

The study included 2,534 older adults with an average age of 75.9 years (SD 3.9) (Table 1). There were slightly more female (52.7%) than male participants, and 52.6% had attained at least a middle school education. Physically frail individuals were older (78.3 vs. 75.1 years), predominantly female (78.9% vs. 44.8%), and more likely to be uneducated (56.7% vs. 12.0%) compared to their physical non-frail counterparts. They had a higher number of medications (45.3% vs. 26.2%) than the physically non-frail. They showed higher depression scores (8.1 vs. 2.0) and lower social participation (1.3 vs. 1.8) than physically non-frail individuals. Physically frail individuals scored lowest and physically non-frail individuals highest in the physical activity environment. Cognitive frailty was observed in older individuals, with an average age of 78.9 years. Cognitively frail individuals were more likely to take five or more medications compared to those taking none (49.3% vs. 31.4%). They exhibited higher depression scores (8.1 vs. 3.0) and lower social participation (1.3 vs. 1.7) than cognitively non-frail individuals. The highest proportion of individuals with social frailty was observed among those receiving medical aid (9.6%, *N* = 44). Individuals with social frailty showed higher depression (4.5 vs. 2.4) and lower social participation scores (1.2 vs. 2.0) than those with social non-frailty.

**Table 1:** Baseline characteristics of physical frailty, cognitive frailty, and social frailty (*n* = 2,534).

Variables	Total (*n* = 2,534)	Physical frailty			Cognitive frailty		Social frailty		
		Non-frail (*n* = 1,356)	Pre-frail (*n* = 1,088)	Frail (*n* = 90)	Non-frail (*n* = 2,465)	Frail (*n* = 69)	Non-frail (*n* = 630)	Pre-frail (*n* = 1,440)	Frail (*n* = 464)
Age (years), mean ± SD	75.9 ± 3.9	75.1 ± 3.6	76.7 ± 3.9	78.3 ± 3.3***	75.8 ± 3.8	78.9 ± 3.6***	75.6 ± 3.9	75.9 ± 3.8	76.3 ± 4.0**
Sex, n (%)				***		**			*
Male	1,199 (47.3)	749 (55.2)	431 (39.6)	19 (21.1)	1,184 (48.0)	15 (21.7)	308 (48.9)	651 (45.2)	240 (51.7)
Female	1,335 (52.7)	607 (44.8)	657 (60.4)	71 (78.9)	1,281 (52.0)	54 (78.3)	322 (51.1)	789 (54.8)	224 (48.3)
Education†, n (%)				***		***			*
Uneducated	507 (20.0)	163 (12.0)	293 (27.0)	51 (56.7)	467 (19.0)	40 (58.0)	100 (15.9)	302 (21.0)	105 (22.7)
Primary school	693 (27.4)	349 (25.8)	324 (29.8)	20 (22.2)	679 (27.6)	14 (20.3)	191 (30.3)	371 (25.8)	131 (28.3)
≥Middle school	1,332 (52.6)	843 (62.2)	470 (43.2)	19 (21.1)	1,317 (53.5)	15 (21.7)	339 (53.8)	766 (53.2)	227 (49.0)
Marital status†, n (%)				**					***
Married	1,728 (68.3)	1,016 (75.0)	664 (61.1)	48 (53.3)	1,689 (68.6)	39 (56.5)	506 (80.6)	932 (64.7)	290 (62.5)
Widowed	740 (29.2)	304 (22.4)	396 (36.4)	40 (44.4)	711 (28.9)	29 (42.1)	119 (18.9)	475 (33.0)	146 (31.5)
Divorced/ separated	61 (2.4)	34 (2.5)	25 (2.3)	2 (2.2)	60 (2.4)	1 (1.4)	3 (0.5)	32 (2.2)	26 (5.6)
Unmarried	3 (0.1)	1 (0.1)	2 (0.2)	0 (0.0)	3 (0.1)	0 (0.0)	0 (0.0)	1 (0.1)	2 (0.4)
Health insurance†, n (%)				***					***
Workplace/local	2,361 (94.9)	1,289 (96.5)	989 (93.0)	83 (94.3)	2,298 (95.0)	63 (94.0)	609 (97.9)	1,340 (95.1)	412 (90.4)
Medical aid	126 (5.1)	47 (3.5)	74 (7.0)	5 (5.7)	122 (5.0)	4 (6.0)	13 (2.1)	69 (4.9)	44 (9.6)
Health literacy†, n (%)				***		**			**
Adequate	555 (22.3)	390 (29.2)	160 (15.0)	5 (5.7)	550 (22.7)	5 (7.4)	161 (26.1)	317 (22.3)	77 (17.1)
Inadequate	1,933 (77.7)	946 (70.8)	904 (85.0)	83 (94.3)	1,870 (77.3)	63 (92.6)	456 (73.9)	1,103 (77.7)	374 (82.9)
Number of Comorbidities (0 ~ 10), mean ± SD	2.3 ± 1.6	2.1 ± 1.6	2.4 ± 1.6	2.6 ± 1.7***	2.3 ± 1.6	2.4 ± 1.5	2.1 ± 1.5	2.3 ± 1.6	2.4 ± 1.7**
Number of medications†, n (%)				***		***			
0–4	1,717 (68.1)	1,001 (73.8)	669 (62.0)	47 (54.7)	1,683 (68.6)	34 (50.7)	431 (68.7)	989 (69.0)	297 (64.6)
≥ 5	804 (31.9)	355 (26.2)	410 (38.0)	39 (45.3)	777 (31.4)	33 (49.3)	196 (31.3)	445 (31.0)	163 (35.4)
Hearing loss†, n (%)				***		**			**
No	1,832 (72.4)	1,025 (75.6)	782 (72.0)	60 (66.7)	1,825 (74.1)	42 (60.9)	472 (75.2)	1,056 (73.3)	304 (65.5)
Yes	700 (27.6)	331 (24.4)	304 (28.0)	30 (33.3)	638 (25.9)	27 (39.1)	156 (24.8)	384 (26.7)	160 (34.5)
Health check-ups†, n (%)				**					***
Yes	2,239 (88.7)	1,226 (90.7)	939 (86.7)	74 (83.1)	2,180 (88.8)	59 (85.5)	569 (90.7)	1,288 (89.8)	382 (82.7)
No	285 (11.3)	126 (9.3)	144 (13.3)	15 (16.9)	275 (11.2)	10 (14.5)	58 (9.3)	147 (10.2)	80 (17.3)
Balance confidence (0 ~ 100), mean ± SD	79.9 ± 19.3	86.5 ± 13.9	74.3 ± 20.0	46.6 ± 23.6***	80.8 ± 18.3	46.8 ± 23.5***	83.6 ± 17.2	80.4 ± 18.5	75.4 ± 21.1***
Fear of falling, n (%)				***		***			*
No	938 (37.0)	624 (46.0)	304 (27.9)	10 (11.1)	932 (37.8)	6 (8.7)	255 (40.5)	520 (36.1)	163 (35.1)
Low	788 (31.1)	459 (33.8)	316 (29.0)	13 (14.4)	779 (31.6)	9 (13.0)	205 (32.5)	445 (30.9)	138 (29.7)
High	808 (31.9)	273 (20.1)	468 (43.0)	67 (74.5)	754 (30.6)	54 (78.3)	170 (27.0)	475 (33.0)	163 (35.2)
Depression (0 ~ 15), mean ± SD	3.1 ± 3.6	2.0 ± 2.7	4.1 ± 3.9	8.1 ± 4.7***	3.0 ± 3.5	8.1 ± 4.5***	2.4 ± 3.1	3.0 ± 3.5	4.5 ± 4.3***
Social participation (0 ~ 8), mean ± SD	1.7 ± 1.0	1.8 ± 1.0	1.6 ± 0.9	1.3 ± 0.8***	1.7 ± 1.0	1.3 ± 0.8***	2.0 ± 0.9	1.8 ± 1.0	1.2 ± 0.9***
Activity environment (16 ~ 65), mean ± SD	51.4 ± 9.2	52.8 ± 8.6	50.1 ± 9.7	47.3 ± 9.2***	51.5 ± 9.2	46.9 ± 8.8***	52.8 ± 9.2	51.3 ± 9.3	50.0 ± 8.8***

### Prevalence of multiple frailty

The physical frailty group comprised 1,356 (53.5%) non-frail, 1,088 (42.9%) pre-frail, and 90 (3.6%) frail individuals at baseline (Supplementary Fig. S1. online). In the cognitive frailty group, there were 2,461 (97.1%) non-frail, and 73 (2.9%) frail individuals. For the social frailty group, the baseline numbers were 630 (24.9%) non-frail, 1,440 (56.8%) pre-frail, and 464 (18.3%) frail individuals.

Among the participants, 369 (14.6%) were classified as non-frail in physical, cognitive, and social dimensions; 621 (24.5%) were pre-frail in physical and social dimensions; and 25 (1.0%) were frail in physical, cognitive, and social dimensions at baseline. For details on other multiple frailty, see Supplementary Table S2.

### Change in frailty status by multiple frailty over time

The changes in frailty status across three dimensions—physical, cognitive, and social frailty—from 2016 to 2017 to 2018–2019 are described (Supplementary Table S3). By the follow-up period, these numbers had shifted to 1,345 (53.6%) non-frail, 1,088 (42.9%) pre-frail, and 77 (3.0%) frail individuals in physical frailty. The changed to 2,429 (95.9%) non-frail and 54 (2.1%) frail individuals in cognitive frailty over time. Social frailty transitioned to 754 (29.8%) non-frail, 1,514 (59.7%) pre-frail, and 266 (10.5%) frail individuals at the time of follow-up.

### Demographic and health-related factors associated with multiple frailty

**Fig. 2:**
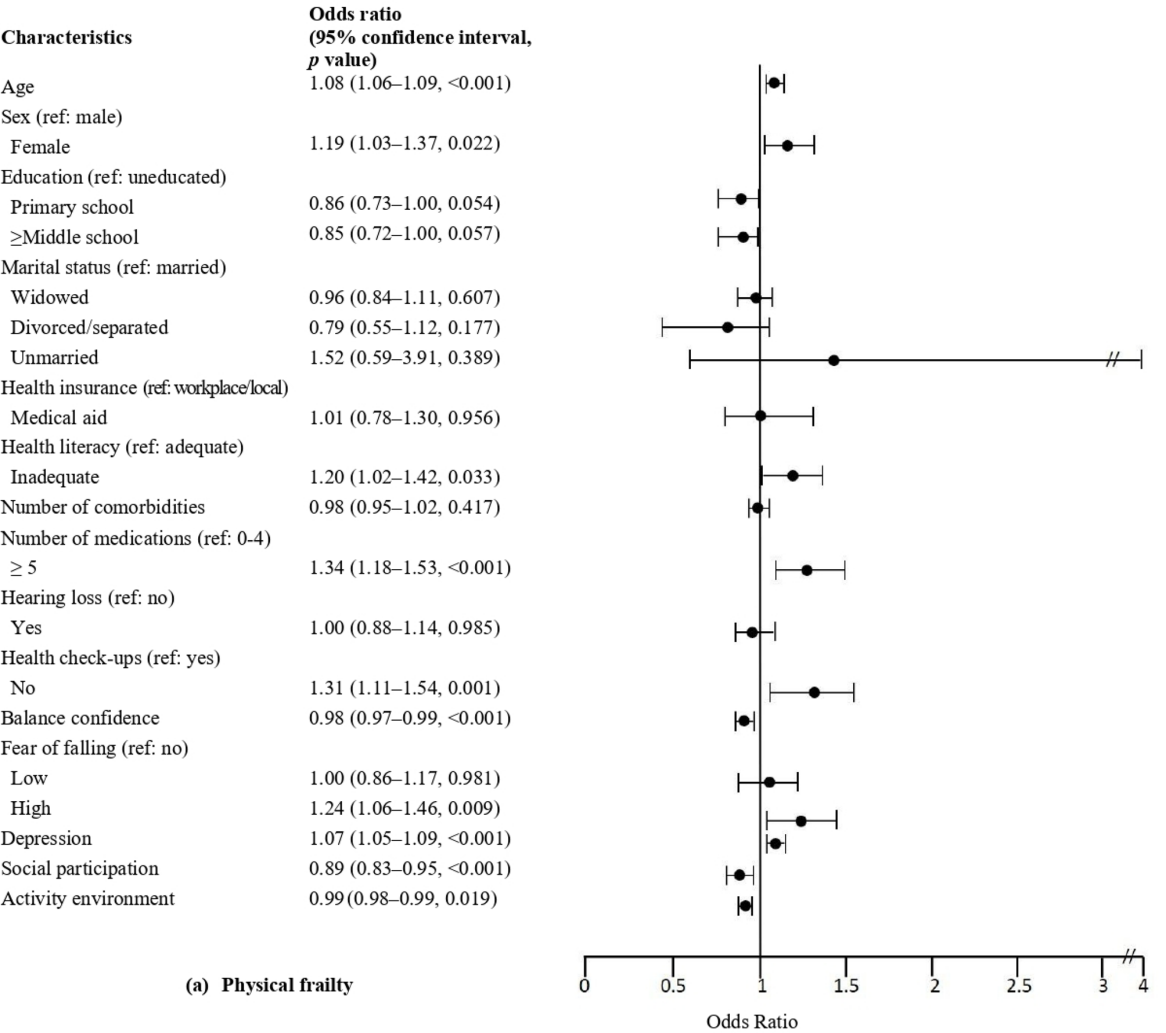

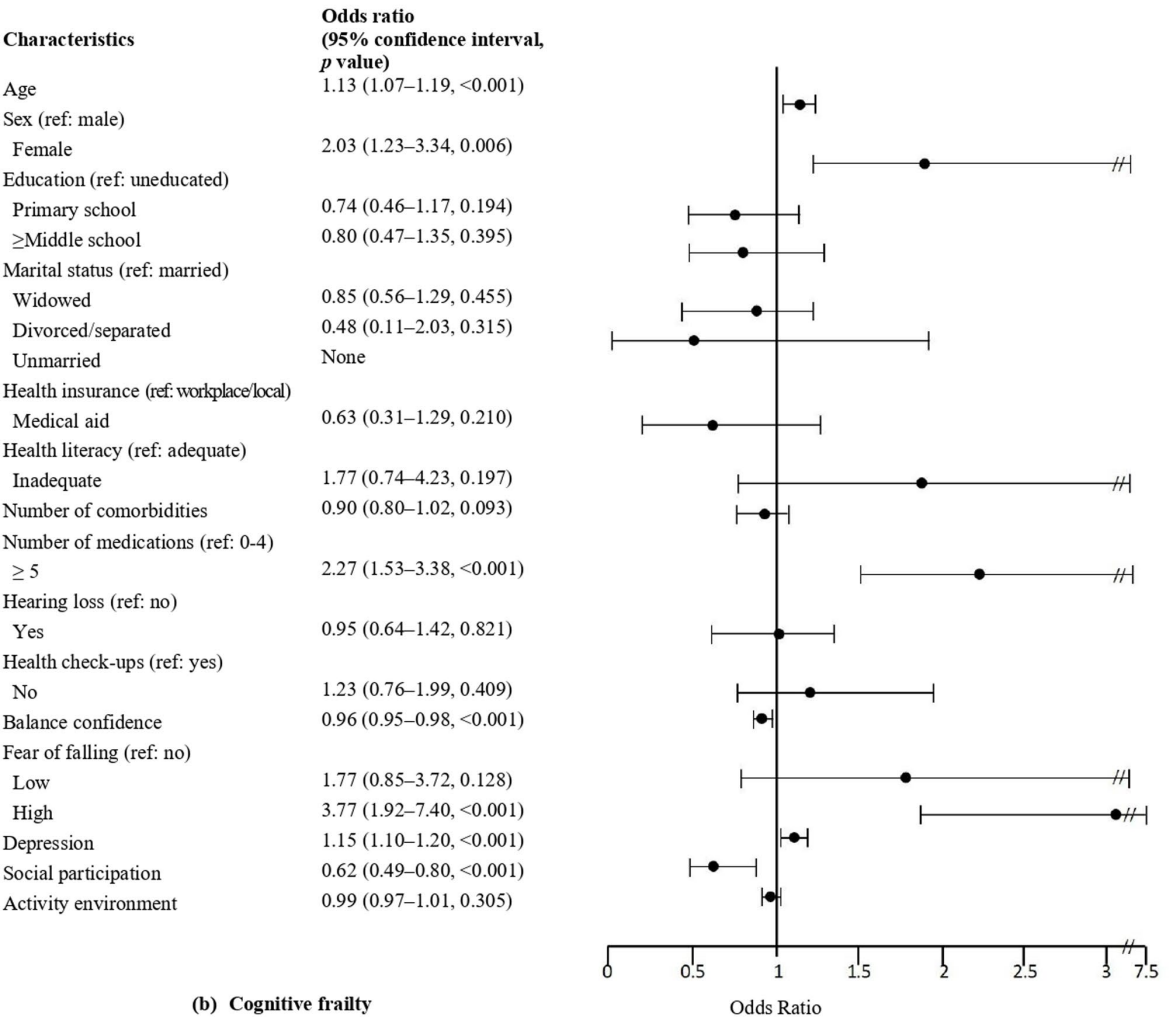

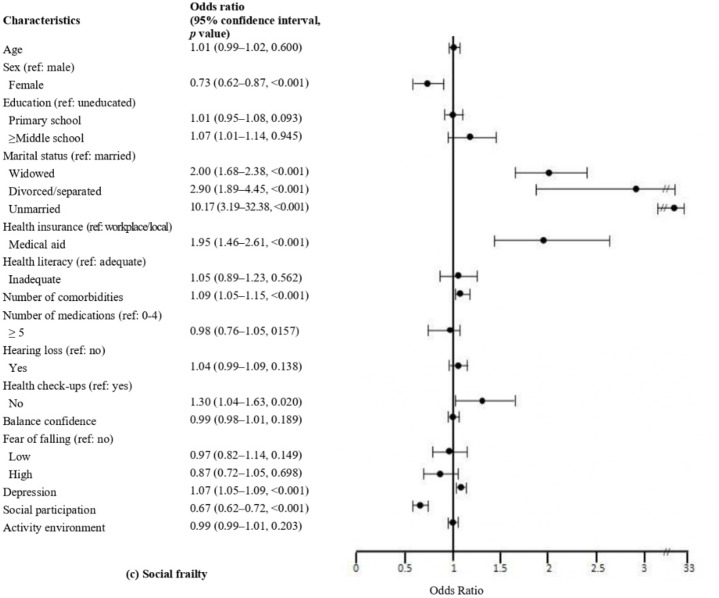
Forest plot association between multiple frailty and characteristics. The black circle indicates the estimated odds ratio, and the horizontal line represents its 95% confidence interval. The vertical line at 1 indicates no association. An odds ratio greater than 1 suggests a positive association, while one less than 1 suggests a negative association.

Frailty can be categorized as single, affecting one specific dimension, or multiple, involving two or more dimensions. Figure 2. shows demographic and health-related factors associated with multiple frailty.

Multiple frailty affected demographic factors. Higher age was linked to a risk of physical and cognitive frailty (OR = 1.08, 95% CI = 1.06–1.09, *p* < 0.001; OR = 1.13, 95% CI = 1.07–1.19, *p* < 0.001, respectively). The ORs for being female in multiple frailty were 1.19, 2.03, and 0.73, respectively (95% CI = 1.03-1.37, *p* = 0.022; 95% CI = 1.23–3.34, *p* = 0.006; 95% CI = 0.62–0.87, *p* < 0.001). Social frailty was significantly associated with being divorced/separated, and widowed individuals compared to being married (OR = 2.00, 95% CI = 1.68–2.38, *p* < 0.001; OR = 2.90, 95% CI = 1.89–4.45, *p* < 0.001, respectively). Regarding marital status, the likelihood of being categorized as social frail was highest among unmarried individuals (OR = 10.17, 95% CI = 3.19–32.38, *p* < 0.001). Participants receiving medical aid showed a significant association with social frailty (95% CI = 1.46–2.61, *p* < 0.001).

Health-related factors were associated with single and multiple frailty. Single frailty, such as a poor physical activity environment, was significantly associated with physical frailty (OR = 0.99, 95% CI = 0.98–0.99, *p* = 0.019), while a higher number of comorbidities was related to a greater likelihood of social frailty (OR = 1.09, 95% CI = 1.05–1.15, *p* < 0.001). For multiple frailty, taking more than five medications increased the odds of physical and cognitive frailty (OR = 1.34, 95% CI = 1.18–1.53, *p* < 0.001; OR = 2.27, 95% CI = 1.53–3.38, *p* < 0.001, respectively), while high activity-specific balance confidence was associated with a lower likelihood of both (OR = 0.98, 95% CI = 0.97–0.99, *p* < 0.001; OR = 0.96, 95% CI = 0.95–0.98, *p* < 0.001, respectively). A high fear of falling was significantly associated with both physical and cognitive frailty (OR = 1.24, 95% CI = 1.06–1.46, *p* = 0.009; OR = 3.77, 95% CI = 1.92–7.40, *p* < 0.001, respectively). The absence of national health check-ups was recognized as a significant correlate for physical and social frailty (OR = 1.31, 95% CI = 1.11–1.54, *p* = 0.001; OR = 1.30, 95% CI = 1.04–1.63, *p* = 0.020, respectively). Depression was identified as having a significant association with multiple frailty (physical frailty OR = 1.07, 95% CI = 1.05–1.09, *p* < 0.001; cognitive frailty OR = 1.15, 95% CI = 1.10–1.20, *p* < 0.001; social frailty OR = 1.07, 95% CI = 1.05–1.09, *p* < 0.001, respectively). Lower social participation was associated with linked to increased odds of multiple frailty (physical frailty OR = 0.89, 95% CI = 0.83–0.95, *p* < 0.001; cognitive frailty OR = 0.62, 95% CI = 0.49–0.80, *p* < 0.001; social frailty OR = 0.67, 95% CI = 0.62–0.72, *p* < 0.001, respectively).

### Factors associated with single and multiple frailty

Single frailty was identified only in health-related factors: physical activity environment was factors of physical frailty, while the number of comorbidities was a factor of social frailty. Multiple frailty was observed, with higher age, taking more than five medications, activity-specific balance confidence, and fear of falling associated with both physical and cognitive frailty. Being female, and the absence of national health check-ups related physical and social frailty. Depression and low social participation were predictors of multiple frailty.

## Discussion

The longitudinal study utilized the KFACS to analyse 2,534 participants. The objectives were to explore the changes in multiple frailty, to identify factors associated with single or multiple frailty, and to compare these factors across the multiple frailty. Health-related factors contributing to physical and cognitive frailty included the number of medications and reduced balance confidence. The absence of national health check-ups was identified as a common new Indicator of physical and social frailty. Depression and lower social participation were significantly associated with multiple frailty. This study offers comprehensive knowledge on associated factors and insights into multiple frailty intervention strategies.

Multiple frailty is not a static condition over time; it is dynamic. The findings demonstrate that an individual can exhibit physical, cognitive, and social frailty concurrently. Compared to a recent previous study conducted in Japan, the results of this study show that physically frail individuals may experience cognitive, psychological, and social frailty^6^. The findings of this study are supported by a systematic review and meta-analysis based on studies from many Asian and European countries, which reported a 42% prevalence of multiple frailty ^5^. The results on the prevalence of multiple frailty indicate that many older adults are affected by various aspects of multiple frailty, demanding an integrated care strategy. These findings highlight the need for a multidisciplinary team-based comprehensive approach to effectively utilize limited healthcare resources in a super-aged society, given the high prevalence of multiple frailty. It is essential for multidisciplinary teams to focus on the prevention, early detection, and effective management of multiple frailty.

Educational level was not significantly associated with physical frailty in this study, which contrasts with previous studies reporting low educational attainment as a risk factor for frailty^10,11,31^. Health literacy—a construct closely linked to education—was significantly associated with physical frailty. This can be because educational attainment is largely a fixed past indicator, whereas health literacy reflects current capacities to understand and implement health information, thereby linking more directly to physical functioning^32^. This suggests that the ability to understand and apply health information may be more directly related to physical health than formal education alone. While prior research has emphasized education as a component of cognitive reserve^33^, health literacy may serve as a modifiable factor through which education exerts its influence. Therefore, regularly assessing health literacy among older adults and providing educational programs to improve it may help reduce physical frailty risk.

Compared to those who were married, widowed individuals showed higher odds of social frailty. The loss of a spouse may substantially reduce the frequency of daily social interactions, as marital partners often serve as the primary facilitators of social engagement for older adults^34,35^. This effect can be particularly pronounced in Korean society, where social activities and community participation are often family-centered, leaving widowed individuals with fewer opportunities for social connection^34,36^. These findings suggest that widowed older adults should be considered a high-priority group for social frailty screening and community-based social support interventions.

Lifelong unmarried status was identified as a major factor for social frailty. Previous studies, when measuring social frailty, included marital status by spousal presence versus absence or broadly classified it as married versus unmarried characteristics, rarely analysing its specific association with lifelong unmarried status^37^. We acknowledge a potential conceptual overlap between lifelong unmarried status and living alone, which is a component of social frailty. This study is the first to report that lifelong unmarried status is a significant associated factor of social frailty. Marriage provides important resources, including emotional support, family networks, and economic stability. However, lifelong unmarried individuals are highly vulnerable to increased social frailty due to a lack of these sustained relational experiences^31,37^. In a reality where significant care for older adults relies on informal caregivers like spouses or children, lifelong unmarried older adults face a limited informal care system, potentially increasing their reliance on public support^31^. Healthcare policymakers need to address this informal care gap by considering the unique characteristics of lifelong unmarried older adults at high risk of social frailty. In a super-aged society, strategies are needed to enhance the accessibility and diversity of public care services. Community healthcare providers are key to this effort, assessing needs, connecting individuals to resources, and advocating for support to ensure stable care, even without a spouse or children.

Polypharmacy was identified as a multiple factor for both physical and cognitive frailty. Previous studies have established a correlation between frailty and the number of medications taken^38,39^. Despite this evidence, polypharmacy management for frail individuals remains lacking in clinical practice. Healthcare providers, given their direct and continuous patient contact, are uniquely positioned to be the first to identify potential polypharmacy issues and initiate vital medication assessments. Healthcare facilities should prioritize regular medication reviews and deprescribing strategies to minimize the risks associated with physical and cognitive frailty. Previous studies have underscored the importance of routine frailty assessments and medication audits in identifying individuals at high risk^40^. Additionally, implementing integrated care approaches that monitor and optimize medication use can help reduce the burden of polypharmacy and its impact on multiple frailty^39^. Future research is essential to determine the effectiveness of these strategies in improving health outcomes for those experiencing multiple frailty.

Balance confidence was a significant protective factor for both physical and cognitive frailty, whereas fear of falling was associated only with physical frailty. In previous studies, both timed up and go (slowness) and strength test (weakness), which are operational definitions of physical frailty^8,17^, were reported to have significant relationships with balance confidence^41^, and the same results were reported for cognitive frailty^11^. In a literature review study, fear of falling was consistently reported to increase the risk of physical frailty^42^. Balance confidence is thought to positively influence daily activities, thus having a broad positive effect across multiple frailty. However, fear of falling is presumed to have a negative effect restricted to specific physical frailty, by potentially restricting or diminishing daily activities. These findings emphasize the importance of interventions focused on improving balance confidence to reduce multiple frailty in older adults. Healthcare providers, by integrating interventions considering not only physical aspects but also psychological aspects, can manage multiple frailty.

The study indicates that higher depression scores are associated with an increased likelihood of multiple frailty. Depression significantly contributes to the risk of physical, cognitive, and social frailty^11,12,43,44^. Depression in older adults is both a key associated factor and a modifiable condition in the management of multiple frailty. However, due to overlapping symptoms such as fatigue, weight loss, decreased physical activity, social withdrawal, and lethargy^43^, it is clinically challenging to clearly distinguish between depression and multiple frailty, which often coexist in older adults. This inherent complexity underscores the critical need for evidence-based practice in their accurate assessment and diagnosis. Therefore, regular mental health assessments of depression and multiple frailty by healthcare providers are essential. To facilitate the early detection of depression and multiple frailty among older adults, it is crucial to educate not only the older adults themselves but also their families and caregivers on the importance of these conditions and their respective roles.

Frequent social participation has been shown to help prevent multiple frailty. This is supported by previous findings^45,46^, as social participation not only provides cognitive stimulation, emotional support, and opportunities for physical activity, but also motivates individuals to engage in healthier behaviors and routines, and facilitates information exchange. The relationship between social participation and multiple frailty suggests that fostering a sense of community and belonging can lead to improved physical, cognitive, and social health outcomes. This can mitigate the effects of pain, depression, and cognitive decline^45,47^. While the benefits of social participation are globally recognized, its forms and cultural significance can vary across different societies, necessitating culturally sensitive approaches in intervention development. For the management of multiple frailty, healthcare providers play a crucial role in identifying and coordinating culturally sensitive community resources that offer social participation programs. This requires comprehensive competence to consider older adults’ diverse needs, preferences, and cultural contexts, facilitating effective linkages within an interdisciplinary framework.

The limitations were as follows. During the two-year follow-up, 477 of the 3,011 baseline participants (15.8%) were excluded from the final analysis. The primary reason for attrition was incomplete assessment (9.5%), followed by withdrawal of consent (3.2%), loss of contact (1.7%), and death (1.2%), with other reasons accounting for less than 0.1% each. Those excluded were more vulnerable, specifically being older and less educated (Supplementary Table S4). In longitudinal studies of older adults, participants who drop out—including those with incomplete assessments— tend to be older and have poorer health profiles or higher frailty levels at baseline compared to those who remain. This suggests that the observed decrease in frailty prevalence over two years may be partly attributable to the loss of more vulnerable individuals, which could lead to an underestimation of the frailty burden in the community-dwelling population. Therefore, the findings should be interpreted with caution given the potential for healthy survival bias^48^. The purpose of this study was to determine predictors associated with multiple frailty. As a result, depression was found to have a significant impact on multiple frailty. However, this study did not sufficiently consider various psychological aspects of older adults, such as loneliness, stress, low self-esteem, anxiety, and feelings of helplessness, compared to physical aspects. Despite these limitations, the study’s major strength is its longitudinal approach to examining changes in multiple frailty and identifying factors associated with it. This study provides the first integrated information on multiple frailty, offering evidence for developing interventions to improve health-related outcomes in older adults.

## Conclusions

This research emphasizes that depression and low social participation are risk factors for multiple frailty. It identifies the determinants associated with multiple frailty, providing integrated insights for nursing practice. Healthcare providers in this field play a key role in recognizing these determinants and implementing regular assessments. Interventions need to be tailored to the multiple frailty and its associated modifiable factors. This study is significant as it comprehensively provides information on the prevalence, dynamics, and determinants of multiple frailty through a longitudinal analysis in the super-aged era.

## Supplementary Information

Below is the link to the electronic supplementary material.

Supplementary Material 1

## Data Availability

The data that support the findings of this study are available from the Elderly Frailty Research Center upon reasonable request (admin@kfacs.kr).
